# High-Voltage Power Supply for Four-Quadrant Dielectric Elastomer Actuators

**DOI:** 10.3390/s24186080

**Published:** 2024-09-20

**Authors:** Haoyue Xing, Qun Hao, Cancan Yao, Zitong Zhang, Jiafu Li, Yang Cheng

**Affiliations:** 1 Key Laboratory of Biomimetic Robots and Systems, Ministry of Education, Beijing Institute of Technology, Beijing 100081, China; bit_clwy@yeah.net (H.X.); qhao@bit.edu.cn (Q.H.); 18754035994@163.com (C.Y.); 3120230619@bit.edu.cn (Z.Z.); 2 National Key Laboratory on Near-Surface Detection, Beijing 100072, China; 3 Precision Measurement Laboratory, National Institute of Metrology, Beijing 100020, China; lijiafu@nim.ac.cn; 4 Yangtze Delta Region Academy of Beijing Institute of Technology, Jiaxing 314003, China

**Keywords:** dielectric elastomer actuators, electric amplifier, high voltage, DC-DC converter

## Abstract

Dielectric elastomer actuators (DEAs) are emerging as promising candidates for various applications in robotics and optical devices due to their lightweight, miniaturization potential, high energy density, simple structure, and low power consumption. However, their effective actuation always demands sophisticated high-voltage driving circuits that are compact and responsive. DEAs need to be capable of generating intricate high-voltage waveforms or simultaneously controlling multiple quadrants with distinct high-voltage levels. This paper proposes a high-voltage power supply for DEAs, featuring a four-quadrant high-voltage driving circuit. The circuit is capable of independently generating high-voltage signals ranging from 100 V to 6000 V and producing arbitrary waveforms with adjustable frequencies. The independent operation of the quadrants without crosstalk showcases the system’s integration and potential for cross-disciplinary applications.

## 1. Introduction

Dielectric elastomer actuators (DEAs) have recently received considerable attention from researchers, as they have a simple structure but high energy density [1], low power consumption (on the order of mW) [2], and high deformation capacity (≥100%). DEAs have demonstrated their potential in various applications due to these advantageous characteristics. One significant application is the field of soft robotics [3,4,5], where their high deformation capacity enables the creation of robots capable of intricate movements, mimicking biological organisms, such as the driving wings of tiny flying robots [6,7] and the fins of fish robots [8], and natural muscles [9,10]. Moreover, DEAs have found utility in tunable optics [11,12,13] and sensors [14], broadening their potential applications.

**Figure 1:**
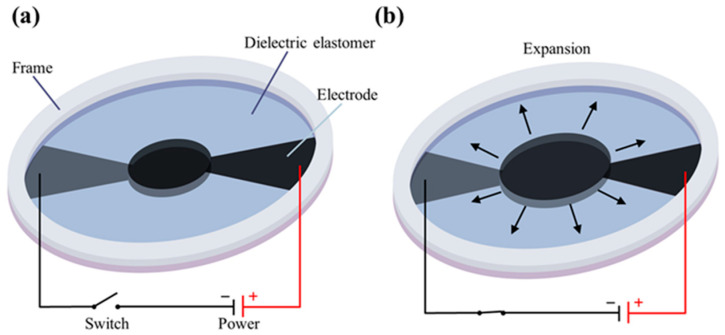
Dielectric elastomer actuator’s working principle and structure: (**a**) initial state and (**b**) expanding state after applying high voltage.

**Figure 2:**
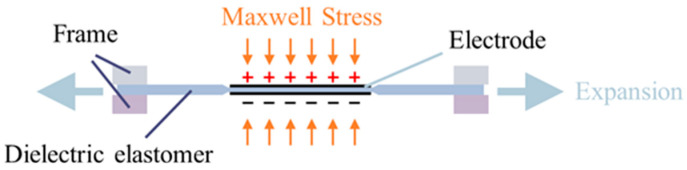
Maxwell stress action when a high voltage is applied.

DEAs usually comprise two flexible electrodes, mainly composed of carbon powder and graphite [15], with a flexible film sandwiched between them, as shown in Figure 1. When voltage is applied to the electrodes, opposite charges accumulate on each electrode, inducing Maxwell stress in the thickness direction. This stress compresses the flexible film between the electrodes, causing the incompressible film to expand in all directions, as depicted in Figure 2. Such expansion can lead to a large strain exceeding 100%. The calculation of Maxwell stress follows the following formula:

(1) $$P=\varepsilon_{0}\varepsilon_{r}\frac{V^{2}}{d}$$

where *P* represents Maxwell stress, *ε*_0_ denotes the dielectric constant of vacuum, *ε_r_* signifies the dielectric coefficient of the elastomer, *V* is the voltage, and *d* indicates the thickness of the elastomer. According to Equation (1), the deformation induced by the elastomeric material is directly proportional to the square of the applied voltage, typically around 100 V/μm. Considering that the thickness of the dielectric elastomer film is generally in the range of tens of micrometers, achieving a sufficiently significant deformation requires applying voltage at the kilovolt level, despite the low power requirement. Generating high voltages above the kilovolt level generally requires a relatively large circuit. However, such a setup is challenging for certain applications of DEAs, such as tiny flying robots, making the miniaturization of DEA driving circuits essential. Furthermore, DEAs are often required to generate complex, controllable waveforms under specific conditions, such as precisely actuating artificial muscles or synchronizing the movement of multiple wings on flying robots. This necessitates a high-voltage power supply capable of delivering arbitrary waveforms and an integrated circuit design that supports multiple quadrants without crosstalk. Meeting these demands introduces a challenge to the development of DEA driving circuits.

Unfortunately, there is currently no available commercial product that meets all the necessary features. High-voltage power supplies commonly used in laboratories are bulky, heavy, and not portable. Researchers have made numerous efforts to address this challenge and develop appropriate high-voltage power supplies. Reference [16] utilized commercial high-voltage converters to create open-source portable high-voltage power supplies, offering cost-effectiveness and capability of delivering high voltages exceeding the kilovolt level. In scenarios that require multi-channel drives, connecting multiple drive boards in series will occupy more volume and complicate the structure. References [17,18] discuss three designs of miniaturized high-voltage drive circuits, employing integrated high-voltage converters or custom high-voltage amplification structures, respectively. These designs can output four channels of variable high-voltage square-wave drive signals. However, the independence of these channels is limited because they share the same high voltages. Consequently, if different types of dielectric elastomer films with distinct breakdown voltages or thicknesses are utilized in the device, it may lead to breakdown issues. Reference [19] developed a low-cost high-voltage converter utilizing the Cockcroft-Walton circuit. However, this circuit occupies a significant amount of space. Some researchers have proposed models aimed at converting low voltage into high voltage [20,21,22,23,24,25], but they have not addressed the challenge of multi-channel driving.

To meet the diverse characteristics required of DEA driving circuits, this paper introduces a four-quadrant high-voltage power supply for DEAs. Each quadrant is capable of independently generating high voltages ranging from 100 to 6000 V. With simple instructions, the system can generate a wide array of waveform shapes. Each quadrant operates autonomously without mutual interference. The high-voltage discharge rate imposes certain constraints on the waveform generation. Specifically, the voltage difference between adjacent sampling points must adhere to a particular relationship to maintain the integrity of the waveform:

(2) $$\Delta V\leq \frac{V_{SR}}{N\times f}$$

where Δ*V* denotes the voltage difference between successive sampling points, *V_SR_* signifies the slew rate of the circuit (discharge rate), *N* is the total number of sampling points per cycle, and *f* corresponds to the waveform frequency. Previous tests have demonstrated that at the 200th sampling point, the system can generate any waveform within the output voltage range at frequencies ranging from 0.1 Hz to 0.5 Hz. The compact design of the entire circuit measures approximately 80 × 80 × 50 mm^3^, ensuring portability and ease of integration. Moreover, it includes an intuitive user interface for generating common waveforms, enhancing accessibility and usability.

## 2. Materials and Methods

The proposed power supply involves two separate printed circuit board (PCB) components, which are independently responsible for signal generation and high-voltage amplification functions. The primary function of the signal generation part is to produce adjustable low-voltage signals, while the high-voltage amplification part amplifies and processes these signals. The advantages of this design include the following: (1) safety and isolation: by physically segregating high-voltage and low-voltage signals, this design effectively mitigates potential maloperations during use, thus safeguarding low-voltage circuits against potential high-voltage damage. This isolation strategy not only enhances system security but also bolsters the stability and reliability of each component. (2) Flexibility and scalability: the autonomy of the high-voltage amplification part provides significant flexibility for the system’s application scenarios. Depending on specific requirements, the signal generation part can be replaced to integrate a low-voltage signal source that is better suited to particular application needs. This modular design approach greatly expands the system’s versatility and facilitates future technological upgrades and functional expansions. These two parts are connected with wiring terminals.

### 2.1. Signal Generation

**Figure 3:**
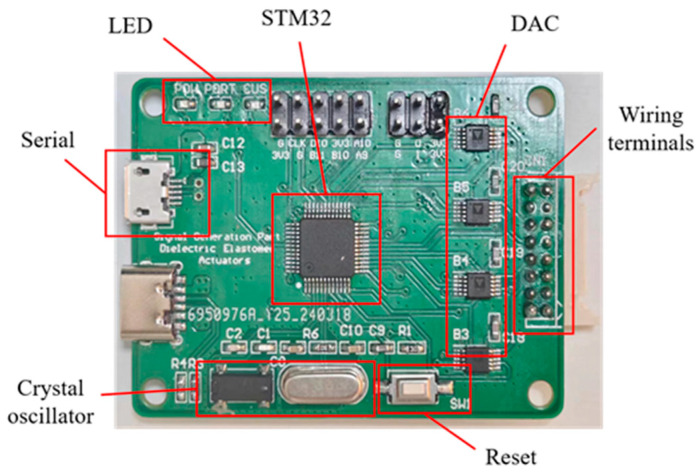
PCB of signal generation part.

The signal generation component in this design is constructed using a printed circuit board (PCB), as depicted in Figure 3, which can receive control signals from a personal computer through a serial communication interface (UART). The signal generation process involves integrating a complete STM32 microcontroller minimum system (STMicroelectronics, Geneva, Switzerland) into the PCB, which is responsible for processing the incoming data signal. Upon receiving the data, the STM32 microcontroller analyzes the data and generates the corresponding analog voltage signal through a digital-to-analog converter (DAC). To ensure independent operation, the system is equipped with four discrete DAC channels, each capable of outputting waveform signals across a scale of 0 to 5 V. Within this range, the voltage is effectively amplified from a lower threshold of 0.7 V, as signals below this level cannot be linearly scaled by the subsequent voltage amplification modules. By altering the data signal transmitted to the STM32, high-voltage waveforms can be generated to meet complex control requirements. Wireless interfaces are reserved on the circuit board, allowing the integration of wireless serial communication modules, such as Bluetooth or Wi-Fi to serial port adapters, for wireless control capabilities in the future. These modules necessitate a 3.3 V power port, a grounding port, and dedicated data transmission and reception ports. The seamless implementation of these wireless functionalities is achieved through the STM32 microcontroller’s integrated serial communication peripherals. Furthermore, the PCB of the signal generation component features three status indicator lights positioned in the upper left corner. These indicator lights serve to indicate the operational status of the power supply, serial communication interface, and DAC. By providing users with intuitive feedback on the system’s status, these indicator lights facilitate system monitoring and debugging processes.

An alternative approach to achieving controllable analog voltage generation involves using a microcontroller unit (MCU) to output pulse-width modulation (PWM) signals, which are then processed through low-pass filters to derive the analog voltage. In most application scenarios, low-pass filters may not eliminate high-frequency components from PWM signals, leading to a certain level of ripple in the filtered analog voltage. Consequently, the filtered analog voltage may not be as precise and smooth as directly utilizing the signal output from a DAC. Furthermore, the inclusion of low-pass filters adds extra hardware complexity, which hampers system integration and efforts toward miniaturization. In systems aiming for high-density integration and compact design, the utilization of a DAC proves more suitable due to its capability to directly generate analog signals. Considering these aspects, a DAC offers significant advantages in signal generation accuracy and circuit design simplicity. It can directly produce high-resolution and low-noise analog signals without requiring additional filtering processing, thereby simplifying the circuit design and improving the overall system performance.

The MCU receives data packets from the computer through the UART. These packets are parsed to extract waveform data for the four independent channels. The MCU will calculate the voltage value for each sampling point based on the parsed data and the specified number of sampling points. The corresponding channel voltage values are cyclically sent to the DAC to generate a low-voltage waveform. Alternatively, the voltage values for each sampling point in the cycle can be pre-calculated and transmitted to the MCU, allowing it to bypass the calculation step and directly send the data to the DAC.

**Figure 4:**
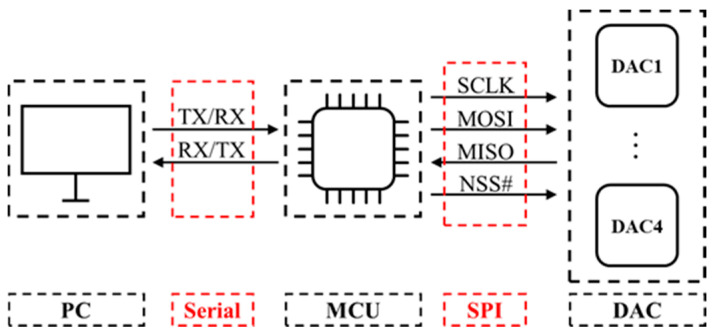
Signal transmission in signal generation part.

Commonly used DAC communication methods include Serial Peripheral Interface (SPI), Inter-Integrated Circuit (I2C), and PWM. This study utilized a single-core STM32 MCU and employed a real-time operating system (RTOS) to simulate a multicore processing environment, optimizing multitask scheduling to achieve the independent control of four DAC channels. While the introduction of an RTOS may lead to minor timing errors, it is essential for enhancing system performance and minimizing communication delays. This requires maximizing the communication rate between the MCU and DAC. Among these communication protocols, SPI stands out for its efficient data transmission rate and multipoint communication capability. SPI allows the concurrent control of multiple secondary devices via a single bus, offering an optimal solution for achieving multi-channel independent control of DACs when hardware resources permit. Therefore, the SPI communication protocol is prioritized to achieve high-speed and independent control of the DAC, as shown in Figure 4.

### 2.2. High-Voltage Amplification

**Figure 5:**
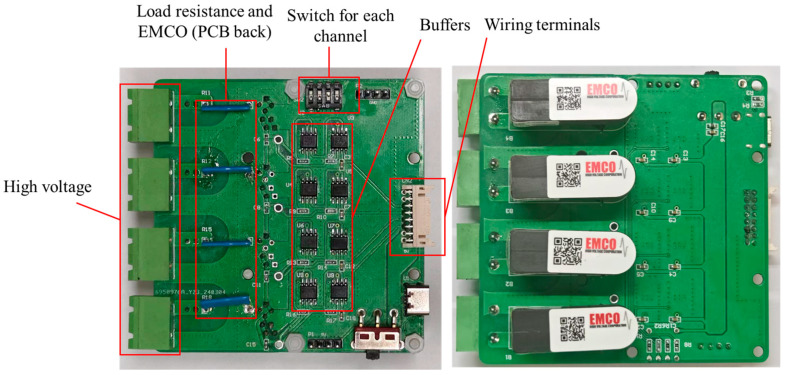
PCB of high-voltage amplification part.

To achieve the goals of integrating and miniaturizing the driving circuit, this study implemented an efficient integrated DC-DC conversion scheme within the voltage amplification section. The EMCO converter (A60P-5 model) from XP Power Company (Tai Seng, Singapore), which is a series DC-DC converter, was chosen for its ability to amplify low-voltage input from 0.7 to 5 V by a factor of 1200, thereby achieving high-voltage output. This selection aims to reduce the number of components while ensuring high-voltage output performance. The PCB in the voltage amplification part (depicted in Figure 5) receives four signals from the signal generation part via wiring terminals. Before transmission to the EMCO converter, these signals undergo isolation and stabilization through a buffer to preserve signal integrity and stability. The inclusion of a buffer aims to improve the signal driving capability, allowing the processed signals to directly power the EMCO, thus facilitating efficient and stable high-voltage signal output.

**Figure 6:**
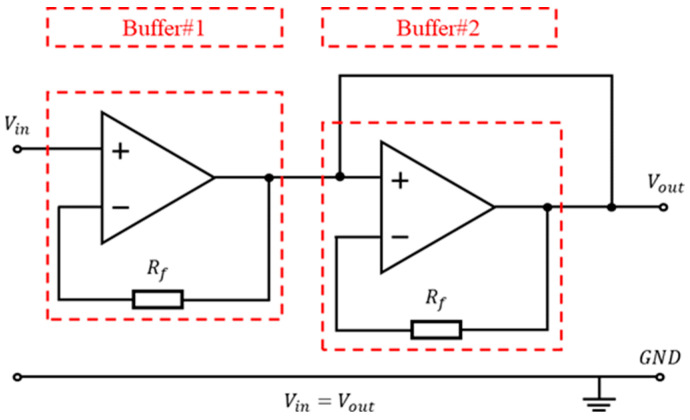
Two voltage followers cascaded to form the buffer.

While the power consumption required to drive DEAs remains relatively low, an EMCO converter requires a certain amount of power to sustain its operation. Direct utilization of the voltage output from the DAC to drive the EMCO may result in a decrease in signal amplitude due to inadequate driving capability. To address this concern, this study introduced a buffer amplifier to enhance the driving capability of the signal. In electronic circuit design, various methods exist to enhance the voltage driving capability, such as voltage followers, emitter followers, and push–pull amplification circuits. Given the presence of internal conduction resistance within transistors and resistors, these components may affect signal amplitude during amplification. To uphold signal integrity, this study used a voltage follower to configure the buffer amplifier (shown in Figure 6).

The voltage follower circuit is designed around a standalone operational amplifier, where the input signal is presented to the positive (non-inverting) input terminal, and the negative (inverting) input is tied to the output terminal. The operational amplifier’s principle of negative feedback ensures that the input and output voltages are nearly identical, with the output precisely tracking the input. Additionally, the operational amplifier’s high input impedance means it draws minimal current from the input signal, thus maintaining the signal’s original state without degradation. Considering the power demands, especially under extreme conditions that may require up to 2.5 W from the DC-DC converter, a single operational amplifier might not offer adequate power delivery capabilities. To overcome this limitation, the system employs a dual operational amplifier configuration (TLV4110 model from Texas Instruments, Dallas, TX, USA). By cascading two of these amplifiers, each voltage follower is capable of supplying approximately 1.5 W, effectively doubling the power output and fulfilling the system’s stringent power requirements.

In the process of driving the DEAs in EMCO converters, it is imperative to connect appropriate load resistors in parallel to discharge accumulated charges during the driving process, as shown in Figure 6. The choice of load resistance significantly affects system performance. A higher load resistance slows down the discharge rate, causing a delay in transitioning the output square-wave signal from high to low levels, thus extending the drop time. Conversely, smaller load resistors can expedite charge release speed and enhance waveform signal accuracy. However, they may escalate current demand, potentially exceeding the EMCO converters’ driving capacity range. Hence, optimizing the selection of load resistance is critical to ensuring effective DEA operation by EMCO converters and maintaining waveform stability. In design, achieving a balance in load resistance size is essential to meet EMCO converters’ maximum driving capacity while minimizing resistance value to enhance response speed and waveform signal stability. Through meticulous calculation and experimental validation, this study determined the optimal load resistance value. This enables effective DEA driving and waveform signal optimization without exceeding the EMCO converters’ capacity.

## 3. Results

In this study, the type-C interface was used to provide power to the driver board, and successful verification was conducted to demonstrate that the designed driving circuit can autonomously generate high-voltage signals ranging from 100 V to 6000 V for each quadrant. The simple instructions can enable the system to generate controllable high-waveform waveforms, which were subsequently tested in practical applications with DEAs. These experiments confirmed the viability of the proposed scheme through rigorous real-world driving tests. To enhance user experience, an intuitive user interface was developed, empowering users to easily control the output of standard waveforms through simple commands. This streamlined operational process significantly improves the ease of conducting experiments and enhances the overall usability.

**Figure 7:**
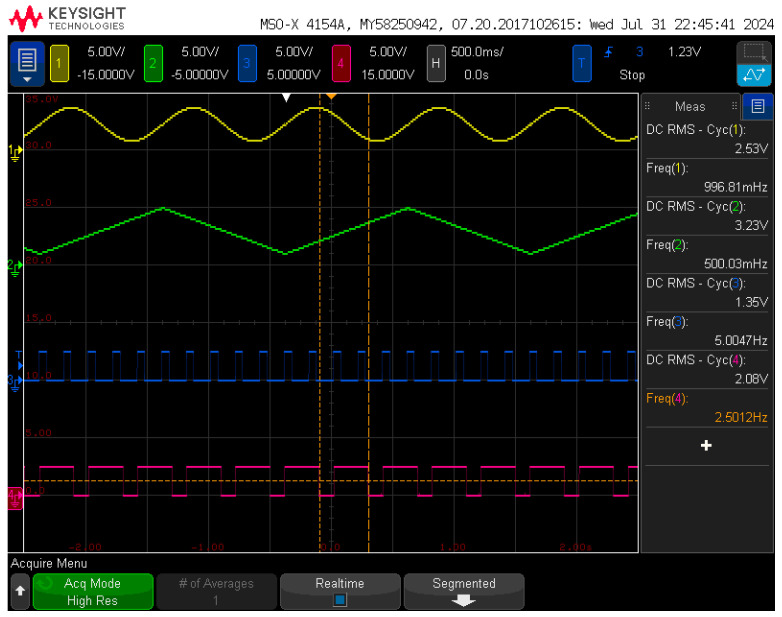
The four output waves of the signal generation component with (1) sine wave; (2) triangular wave; (3) square wave with 30% duty; and (4) square wave with 80% duty.

Figure 7 illustrates the capability of the signal generation component in the circuit board to produce independent signals across the four quadrants, which were captured using the high-resolution mode of an oscilloscope. Each quadrant’s signal was sampled at 200 points per period. The yellow trace represents a sine wave, oscillating between a peak voltage of 4 V and a trough voltage of 1 V. The calculated DC effective value is approximately 2.58 V, with a frequency of 1 Hz. The measured outcomes closely align with the theoretical predictions, with any voltage discrepancies attributed to the signal fitting inaccuracies from the stepped waveform. The green trace corresponds to a triangular wave, with voltages ranging from a maximum of 5 V to a minimum of 1 V. The theoretical DC effective value for this waveform was estimated to be 3.21 V, operating at a frequency of 0.5 Hz, and the actual measurements were found to be in substantial agreement with the theoretical values. The blue trace depicts a square wave with a 30% duty cycle at a frequency of 5 Hz, while the purple trace shows another square wave with an 80% duty cycle and a frequency of 2.5 Hz, featuring a high voltage of 5 V and a low voltage of 0 V. The measurements for these signals are as anticipated. At the time of these tests, the signal amplification stage was not engaged, resulting in an ideal representation of the waveform signals. This confirms the efficacy of the signal generation component in producing accurate and reliable waveforms without the influence of amplification errors.

**Figure 8:**
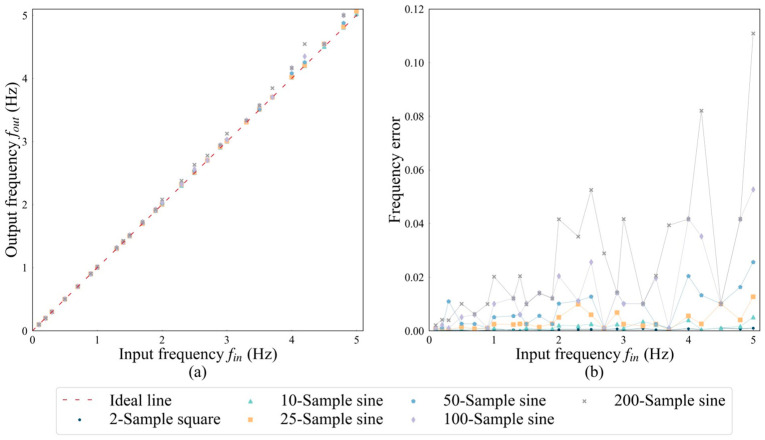
The variation in output frequency with input frequency at different sampling frequencies: (**a**) measured values and (**b**) percentage of error.

The system’s frequency error can be attributed to two primary sources. The initial source is associated with the data transmission and parsing process from the STM32 microcontroller to the DAC, which introduces a latency of approximately 35 microseconds. While this delay can be partially compensated for by adjusting the intervals between sampling points, perfect accuracy cannot be guaranteed. The resultant errors manifest in two main ways: firstly, they manifest as an increase in the output frequency above the target frequency, as depicted in Figure 8a, where the measured points consistently deviate above the ideal line. Secondly, as the number of sampling points per cycle increases, the duration of data transmission and processing lengthens, thus amplifying the errors, as illustrated in Figure 8b. The second source of errors stems from the precision loss incurred by the internal floating-point arithmetic of the STM32 microcontroller and the RTOS clock granularity. In Figure 8b, despite varying the sampling frequencies, a consistent error is observed at certain frequencies, exemplified by the 2.7 Hz data point where the error is uniform across different curves. The system shows fewer errors at lower sampling frequencies and corresponding input frequencies. As the input frequency increases, the error tends to amplify. Specifically, with a configuration of 200 sampling points within a single cycle at an input frequency of 5 Hz, the error peaks at around 11%.

**Figure 9:**
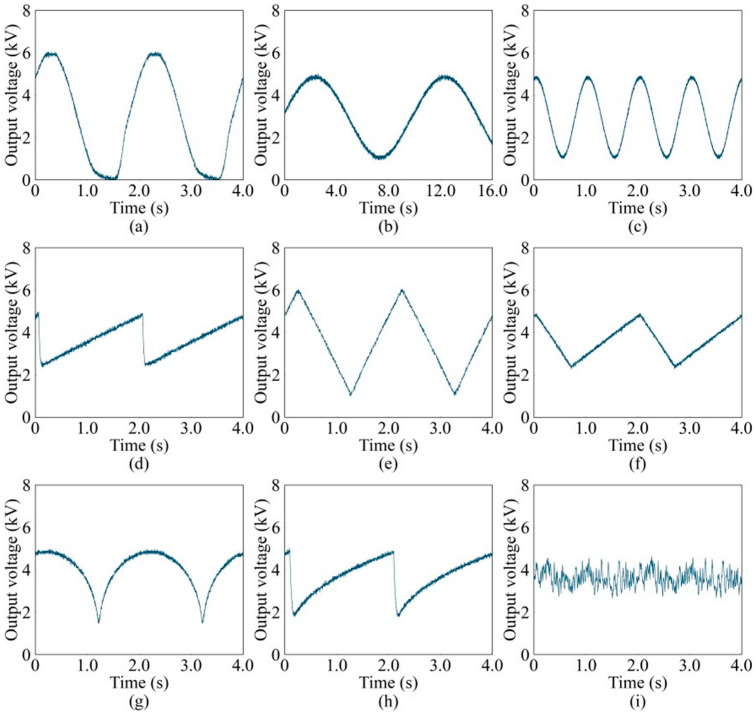
High-voltage waveform signal: (**a**) 0–6 kV sine wave at 0.5 Hz, (**b**) 1–5 kV sine wave at 0.1 Hz, (**c**) 1–5 kV sine wave at 1 Hz, (**d**) 2–5 kV triangular wave with full rise at 0.5 Hz, (**e**) 1–6 kV triangular wave with 50% rise at 0.5 Hz, (**f**) 2–5 kV triangular wave with 70% rise at 0.5 Hz, (**g**) 1–5 kV semi-circular wave at 0.5 Hz, (**h**) 2–5 kV semi-parabolic wave at 0.5 Hz, and (**i**) stochastic noise wave.

By integrating the low-voltage signals from the signal generation component with the voltage amplification component, the system can produce high-voltage signals, as demonstrated in Figure 9. The captured waveforms represent a detailed profile with 200 sampling points for one cycle. Through sending waveform data or pre-calculated sampling point voltages, a diverse array of high-voltage signals is output, encompassing sine waves of varying frequencies (Figure 9a–c), triangular waves with distinct rise times (Figure 9d–f), semi-circular arc waveforms (Figure 9g), semi-parabolic waveforms (Figure 9h), and stochastic noise signals (Figure 9i). Specifically, the sine wave in Figure 9a exhibits some distortion at the lower voltage extremities, which is attributable to the signal’s minimum voltage level reaching 0 V. When such low-voltage inputs are fed into the voltage amplification stage, they are not amplified linearly, leading to this distortion. Despite this, the system maintains its ability to generate accurate high-voltage waveforms within the operational voltage limits, ensuring suitability for a multitude of complex and demanding applications.

**Figure 10:**
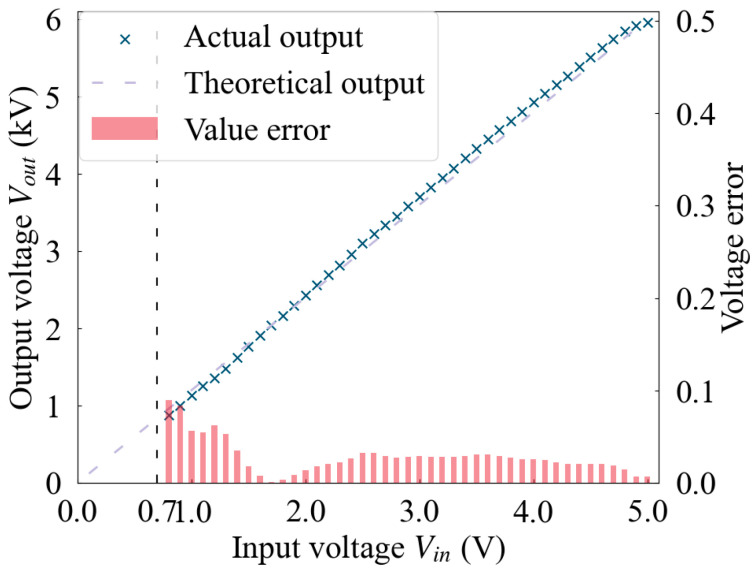
Voltage response and error of the driving circuit.

In the context of DC high-voltage signal output, this study meticulously examined the correlation between the actual high-voltage levels produced and their theoretical counterparts, as depicted in Figure 10, thereby quantifying the error. For inputs within the low-voltage signal range of 0.7 V to 5 V, the resultant high-voltage output was found to be largely in alignment with theoretical expectations. Within the 0.7 V to 1.3 V bracket, the error is approximately between 5% and 10%, whereas for the remaining range, it is noted to be less than 5%. The primary contributor to the high-voltage error was identified as the buffering effect on the low-voltage signal, which introduces noise that, upon amplification, escalates to the order of tens of volts.

**Figure 11:**
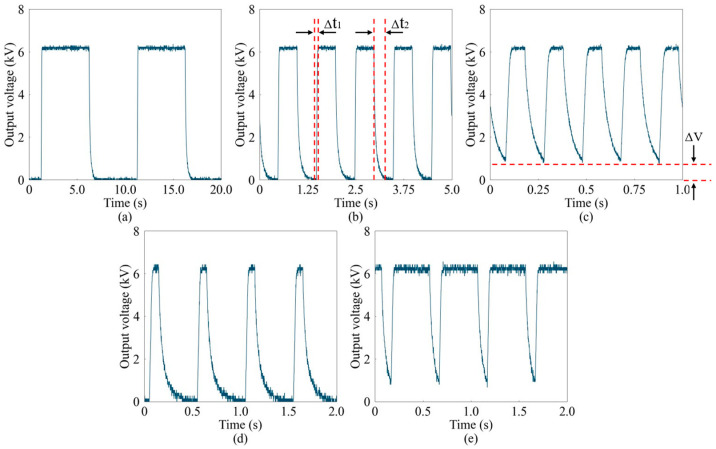
Waveform diagrams of different square-wave signals at a high voltage of 6 kV. (**a**–**c**): 0.1 Hz, 1 Hz, and 5 Hz with 50% duty. (**d**,**e**): 2 Hz with 20% duty and 80% duty.

In practical applications, high-voltage signals are subject to specific constraints, as defined by Equation (2). Figure 11 displays the results of the tests conducted using square-wave signals with varying frequencies and duty cycles, where the high level of the square wave is limited to 6 kV and the low level is 0 V. As the frequency increases, the attenuation time of the square-wave signal also increases. This longer attenuation time is mainly attributed to the challenge of synchronizing the charge release rate with the higher signal frequency during high-frequency operation. Despite carefully selecting the load resistance during circuit design to balance power requirements, the load resistance cannot be set too low, which naturally prolongs the discharge time. Additionally, the internal operations of the DC-DC converter also require a finite discharge period.

**Figure 12:**
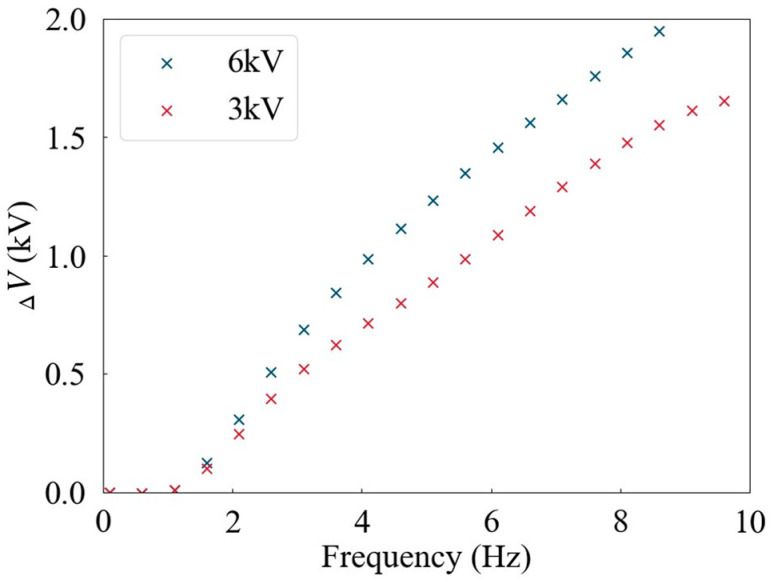
ΔV increases as frequency increases.

The offset voltage Δ*V*, which is the deviation of the low level from 0 V under a square wave with a 50% duty cycle, exhibits an upward trend as frequency increases. To precisely delineate this relationship, an exhaustive test was performed to ascertain the correlation between the offset voltage and frequency. Figure 12 illustrates the offset voltage’s dependence on frequency, plotted as a function of frequency. Consequently, when setting high-voltage waveforms, it is imperative to ensure that the voltage difference between successive sampling points adheres to the stipulations outlined in Formula (2).

**Figure 13:**
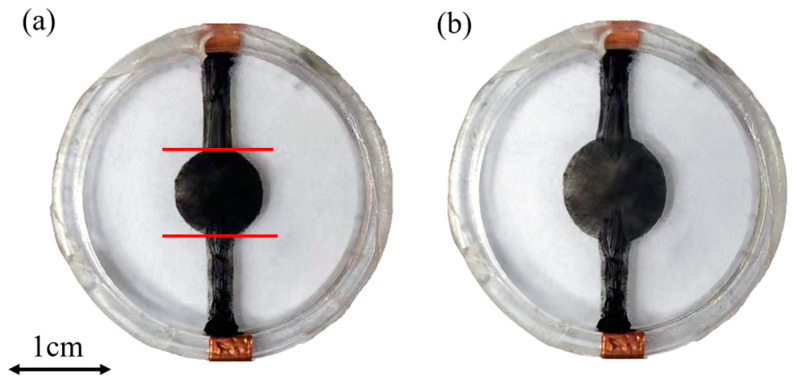
Expansion of dielectric elastomer after applying voltage: (**a**) initial state and (**b**) state after applying 3 kV.

**Figure 14:**
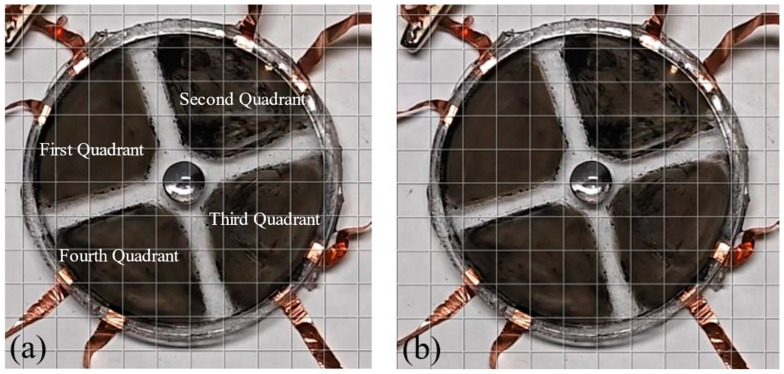
The high-voltage power supply separately or simultaneously drives the four quadrants of the DEA, as shown in Supplementary Video S1 and Supplementary Video S2, respectively. (**a**) Initial state; (**b**) state after applying a square-wave voltage with a frequency of 0.5 Hz and a peak voltage of 3 kV.

This study demonstrated the utilization of a driving circuit to transmit high-voltage signals to a dielectric elastomer actuator. Figure 13 depicts the effect of applying a DC high-voltage signal of 3 kV to the dielectric elastomer, resulting in a significant expansion of the black electrode area in the center compared to its initial state. Figure 14 illustrates the application of this circuit to drive four dielectric elastomer electrodes. In Figure 14a, the electrodes are represented as four quadrants, with each quadrant receiving a square-wave signal at a frequency of 0.5 Hz and a peak voltage of 3 kV. Figure 14b depicts the deformation of the four-quadrant dielectric elastomer under high-voltage actuation. The deformation appears relatively small in the static display because of the large surface area of the compliant electrodes. As shown in the supplementary files, it is clearly observed that the dynamic deformations of the four-quadrant dielectric elastomer fluctuate with the application of square-wave voltages. These experiments validated the effectiveness of the circuit in efficiently driving four-quadrant dielectric elastomers.

**Figure 15:**
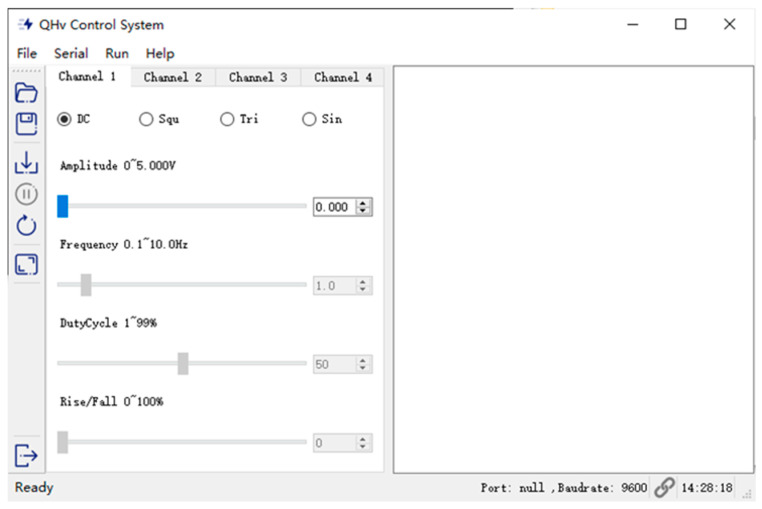
User-friendly interface for controlling the four-quadrant high-voltage output.

To streamline the utilization of common high-voltage waveforms, Figure 15 presents a user-friendly Python-based interface. This interface enables the circuit to output a variety of high-voltage signals, including DC, square wave, sine wave, and triangular wave, with the simplicity of basic commands. For DC, sine wave, and triangular wave, the minimum voltage threshold is set at 100 V. Furthermore, the interface allows the generation of other waveform signals through pre-calculated sampling point voltages. Users can select an appropriate number of sampling points to create sampling data. This data set can then be transmitted to the STM32 microcontroller via the serial port, thereby facilitating the output of complex waveforms.

## 4. Discussion

Existing high-voltage power supplies for dielectric elastomer actuators often depend on commercial or self-built DC-DC converters to attain high-voltage output [16,17,18,19,20,21,22,23,24]. These systems commonly utilize optocouplers or transistors to accurately regulate the voltage levels by adjusting the duty cycle and frequency, generating a PWM (pulse-width modulation) signal for the high-voltage output. This method provides the advantages of generating high-frequency square waves and stable DC high voltages, rendering it appropriate for a broad spectrum of applications, as shown in Table 1.

In contrast, the power supply proposed in this paper takes a new approach by directly modulating the high-voltage output. It features a compact and simple design, facilitating the creation of a wider range of complex and diverse high-voltage waveforms. This flexibility enables it to accommodate a broader spectrum of application scenarios. Nevertheless, this innovation also has some limitations, especially in high-frequency operations, where frequency restrictions and the resulting high costs present significant challenges.

A key aspect of this paper is that the cost of commercial DC-DC converters, which accounts for over 90% of the total device cost in the proposed high-voltage power supply, could hinder its widespread adoption. These issues imply the need for future research to focus on the development and optimization of converters. The aim is to improve their voltage swing characteristics to better meet the demands of high-frequency driving while simultaneously working to reduce costs.

## 5. Conclusions

The actuation of DEAs demands high-voltage driving circuits that are compact and responsive. It is often necessary to generate intricate high-voltage waveforms or simultaneously control multiple channels with distinct high-voltage levels. This paper proposes a high-voltage power supply for four-quadrant DEAs, which combines a signal generation component and a voltage amplification component. The system generates low-voltage signals that can be configured into various waveforms and then amplifies them to a high-voltage range of 100 V to 6000 V, enabling independent and interference-free channel operation. Due to its compact, lightweight design and high flexibility, the proposed power supply is ideal for complex applications, providing a versatile solution for high-voltage signal output.

## Figures and Tables

**Table 1:** Comparison of the high-voltage power supplies in the references with that in this paper.

References	Control Signal of Waveform	Number of Voltages	Output Voltage	Output Waveform	Volume or Area	Weight
[16]	PWM	1	0~5 kV	Square/DC	120 × 55 × 25 cm^3^	60 g
[17]	PWM	4	0~2 kV	Square/DC	5024 cm^2^	56.52 g
[18]	PWM	3	0~16 kV	Square/DC	/	78 g
[19]	PWM	1	0~4 kV	Square/DC	/	/
[20]	AC voltage	1	0~3.5 kV	/	/	/
[21]	PWM	1	0~1 kV	Square/DC	/	/
[22]	PWM	1	0~600 V	Diversity	/	1.78 g
[24]	PTGD topology	1	0~7 kV	/	/	/
Our work	Voltages calculated by STM32 for each sampling point	4	0.1~6 kV	Diversity	80 × 80 × 50 cm^3^	62 g

## Data Availability

The data that support the findings of this study are available from the corresponding author upon reasonable request.

## Supplementary Materials

The following supporting information can be downloaded at https://www.mdpi.com/article/10.3390/s24186080/s1, Video S1: The high-voltage power supply separately drives the four quadrants of DEAs. Video S2: The high-voltage power supply simultaneously drives the four quadrants of DEAs.
